# Psychodiabetology: The Challenge of the Future?

**DOI:** 10.3390/jcm13082236

**Published:** 2024-04-12

**Authors:** Marta Brzuszek, Maciej Kochman, Artur Mazur

**Affiliations:** 1 Institute of Health Sciences, College of Medical Sciences, University of Rzeszów, ul. Warzywna 1a, 35-310 Rzeszów, Poland; 2 Department of Physiotherapy, Institute of Health Sciences, College of Medical Sciences, University of Rzeszów, ul. Marszałkowska 24, 35-215 Rzeszów, Poland; 3 Institute of Medical Science, College of Medical Sciences, University of Rzeszów, ul. Warzywna 1a, 35-215 Rzeszów, Poland

**Keywords:** diabetes mellitus, type 1, chronic disease, determination of healthcare needs, child, hospitalized, mental health, generations

## Abstract

The number of people suffering from diabetes, including type 1, is constantly increasing both in Poland and worldwide. Type 1 diabetes is a chronic disease characterized by uncertain prognosis and relapses, as well as permanent, irreversible, and progressive changes in health status. The ongoing disease results in dysfunction or disability, and the patient requires specialized supervision, care, and rehabilitation. However, the success of therapy does not depend solely on the perfection of treatment, but also on the patient’s readiness to change their lifestyle and cooperate with the therapeutic team. The patient’s constant alertness in making therapeutic decisions does not always lead to expected treatment results, and the risk of hypoglycemia associated with intensive insulin treatment depletes the patient’s motivation for treatment, leading over time to the development of ‘therapeutic burnout’ and psychiatric disorders. This narrative review is an attempt to summarize the knowledge and possible future solutions in diabetes type 1 in Poland as well as highlight the importance of comprehensive care, including psychological care, which appears fundamental in a chronic disease such as type 1 diabetes. Therefore, the aim of the study was to present generational changes and psychosocial problems of patients with type 1 diabetes and to identify urgent challenges in diabetic care. Attention should be paid to the deteriorating mental condition of the young generations, who, in the course of diabetes, are exposed to additional psychological and psychiatric health problems. The next generation of patients will require more psychological care, which is why the challenge of the future is to create psychodiabetology centers.

## 1. Introduction

The number of patients with diabetes, including type 1 diabetes, is increasing both in Poland and around the world. Data published in 2021 by the International Diabetes Federation (IDF) indicate that there are 537 million people with diabetes in the world, and by 2045, this number will increase by approximately 50% [1,2]. According to data from the IDF Atlas, 1.2 million children and adolescents suffered from type 1 diabetes worldwide in 2021. In the age group 0–10 years, this number increases by 149,500 new cases of diabetes every year [1]. In turn, in the group under 15 years of age, type 1 diabetes is diagnosed in approximately 108,200 children and adolescents each year, and the estimated overall number of patients with type 1 diabetes in this age group amounts to 651,700 [1]. In the ranking of the 10 countries with the largest number of children with type 1 diabetes aged 0–18, India was the first (229.4 thousand), followed by the United States of America (157.9 thousand) and Brazil (92.3 thousand). Also, in these regions, the highest increase in new cases was recorded and amounts to 24,18.2, and 8.9 thousand per year, respectively. The highest incidence rates were recorded in Finland (52.2), Sweden (44.1), Kuwait (41.7), Qatar (38.1), Canada (37.9), Algeria (34.8), Norway (33.6), Saudi Arabia (31.4), Great Britain (28.1), and Ireland (27.5) [1]. Epidemiological data from Poland are characterized by equally high incidence rates. According to data from the Report of the Institute of Health Protection, children with diabetes aged 0–14 amount to 542,000 worldwide, while in Poland there are 6400 cases in the group 0–14 years of age and 180,00 in the group over 14 years of age [3]. Currently, the number of children with diabetes has been increasing. With respect to the division into IDF regions, the incidence of type 1 diabetes in the pediatric population is the highest in Europe (296,500 patients; 31.100 new cases per year) [1]. The European countries with the highest incidence of type 1 diabetes in children (0–14 years) are Finland, Sweden, Norway, and Great Britain, and countries with the highest estimated number of prevalent cases of type 1 diabetes in children and adolescents (0–19 years) per annum are the Russian Federation, Germany, and the United Kingdom [1]. Global trends of the increasing incidence rate of diabetes also affect Poland, which in the 1980s and 1990s was considered a country with a very low incidence rate (6.6/100,000/year) [4]. An unfavorable change was recorded in 2004, when the incidence rate increased approximately 2.5 times and amounted to 17.66/100,000/year [5]. The current incidence rates of type 1 diabetes in Poland reach up to 35/100,000/year [6,7,8].

Type 1 diabetes belongs to a group of metabolic diseases characterized by hyperglycemia resulting from the lack/deficiency of endogenous insulin. Hyperglycemia occurring in the latency period of the disease, before the diagnosis, contributes to the activation of the process of non-enzymatic glycation of proteins, oxidative stress, and modification of LDL particles, the toxic effect of which on the vascular endothelium is a recognized factor in the development of vascular complications [9]. It is one of the most common diseases in the population of children and adolescents with a sudden onset; the disease itself is incurable, and the survival time is approximately 12 years shorter compared to the general population, and it is associated with the occurrence of acute and chronic complications [10]. The development of the disease is multifactorial and, in most cases, is immunologically related to the presence of antibodies specific to this type of diabetes [10]. Genetic, immunological, and environmental factors may also contribute to the development of the disease [10]. The destruction of pancreatic beta-cells, which results in absolute insulin deficiency, requires insulin substitution together with diet and physical activity as the way of treating this disease for the rest of life. In the case of younger children, responsibility for the treatment is taken by parents or carers, and then by the adolescents themselves. A number of variables of the developmental period, such as the rhythm of the day, sleep and wakefulness, body weight and height, energy demand, and secretion of adolescent and pubertal hormones, significantly modulate the need for insulin, and the ability to learn and the ability to think logically, as well as the ability to make therapeutic decisions, determines readiness to cooperate with the therapeutic team [8]. On the way to therapeutic success, patients receive new tools to facilitate self-control. The technological advances that have been, and continue to be, created in insulin therapy methods and glycemic monitoring make it possible to achieve optimal results in the metabolic equilibrium of diabetes, provided that conscious work is undertaken by the whole family in adapting to the chronic disease. The Conceptual Model of Childhood Adaptation to Type 1 Diabetes proposed by Whittemore et al. very aptly depicts the dynamics of factors modulating the psychological well-being of a patient with type 1 diabetes. It indicates the dynamics and complexity of adaptation processes, in which depression can be a strong predictor of neglect of treatment management or a psychological response to diabetes treatment failure [11].

Increasingly improved methods and tools supporting diabetes therapy seem to resonate with the generation of patients belonging to the Z and Alpha generations, the generation of “Always on” children or the Net Gen, a generation that is able to use the Internet to access to information like no other before [12]. Type 1 diabetes is characterized by a long duration, uncertain prognosis as to its course, recurrent nature with relapses, as well as permanent, irreversible, and progressive changes in health status. The ongoing disease process results in dysfunction or disability, and the patient requires specialized supervision, care, and rehabilitation [13].

**Figure 1:**
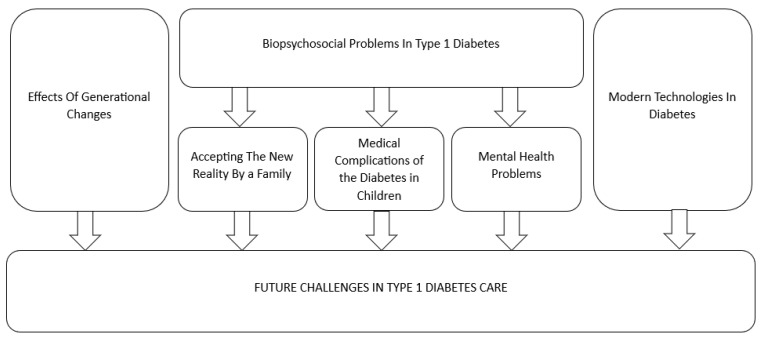
Conceptual model of future challenges in type 1 diabetes care.

This study is an attempt at a narrative review of the knowledge, characteristics of the condition, and possible future solutions in diabetes type 1 in Poland. Therefore, this study aimed to present the generational changes and psychosocial problems of patients with type 1 diabetes and to identify urgent challenges in diabetes care. For this purpose, an analysis of the available literature was carried out in electronic databases and scientific books related to type 1 diabetes management. A literature review involved analyzing, evaluating, and summarizing scientific literature. This review summary diagram has been shown in Figure 1.

## 2. Biopsychosocial Problems in Diabetes

### 2.1. Accepting the New Reality by a Family

In pediatric patients, factors such as an early age at diagnosis and long duration of illness are particularly important factors in the development of chronic complications of diabetes. According to Bronfenbrenner’s ecological concept, a child functions and gets sick in microsystems such as the family, peers, and school class. Upon receiving a diagnosis, the family has to go through all stages of a chronic disease: shock, upheaval, rebellion, denial, and then apparent and ultimately constructive adaptation [14]. The process of becoming ready to accept diabetes takes a long time. According to Nitka-Siemińska, it lasts approximately 9 months in parents and depends on the age at which the child became ill. Six months after the child was diagnosed with diabetes, approximately 24% of mothers and 22% of fathers were diagnosed with full-blown post-traumatic stress disorder. Also, in children newly diagnosed with diabetes, adaptation is slightly shorter than in parents and lasts from 6–9 months [15].

The dynamics of physiological changes during adolescence, which affect the variability of insulin demand, is a source of high and long-term burden for parents in self-control, the ideal of which is the ability to analyze, predict, and make therapeutic decisions in passive and active self-control. Parents of the youngest patients face difficult decisions about resigning from work, which worsens the family’s financial situation and excludes the child from interactions with peers, which is the case in other countries [16]. The difficulty in monitoring acute complications of diabetes in the youngest age group and in those who do not have symptoms of hypoglycemia was recorded in 30% of 656 children with type 1 diabetes, which in turn triples the likelihood of a severe episode of hypoglycemia with seizures and loss of consciousness. Repeated episodes of hypoglycemia may result in a reduction in clinical symptoms and an automatic response to subsequent episodes of hypoglycemia [17,18].

### 2.2. Medical Complications of Diabetes in Children

Striving for normoglycemia carries the risk of hypoglycemia, which impairs intellectual and psychomotor functions, especially in children under 5 years of age due to special susceptibility resulting from the immaturity of the nervous system [19]. The efficiency of the counter-regulatory system decreases with the duration of diabetes due to the passive absorption of insulin into the bloodstream from the site of its administration, reduced ability to secrete glucagon by pancreatic beta-cells, and impaired adrenaline secretion [20]. A weaker response of the counter-regulatory system at night is the cause of very frequent, prolonged hypoglycemia in the pediatric population, occurring for approximately 40% of the night [18]. Children who often experience severe hypoglycemia have poorer verbal skills, poorer short-term memory, and a poorer speed of processing nonverbal information. Repeated episodes of hypoglycemic seizures in young children can cause central temporal sclerosis, found in 16% of children with early-onset diabetes [21]. Recurrent hypo and hyperglycemia contribute to the deterioration of the ability to memorize and learn, and the return of intellectual functions to the state before the incident of severe hypoglycemia may take approximately 1.5 days. At the same time, the plasticity of this system in children creates a chance to replace the disturbed areas of the brain with other areas as a result of a compensation mechanism [22]. Fear of hypoglycemia (FoH) is experienced by both children and parents. It gives rise to protective behaviors against hypoglycemia, with attempts to maintain high blood glucose concentrations, adversely affecting metabolic compensation [23,24,25].

**Figure 2:**
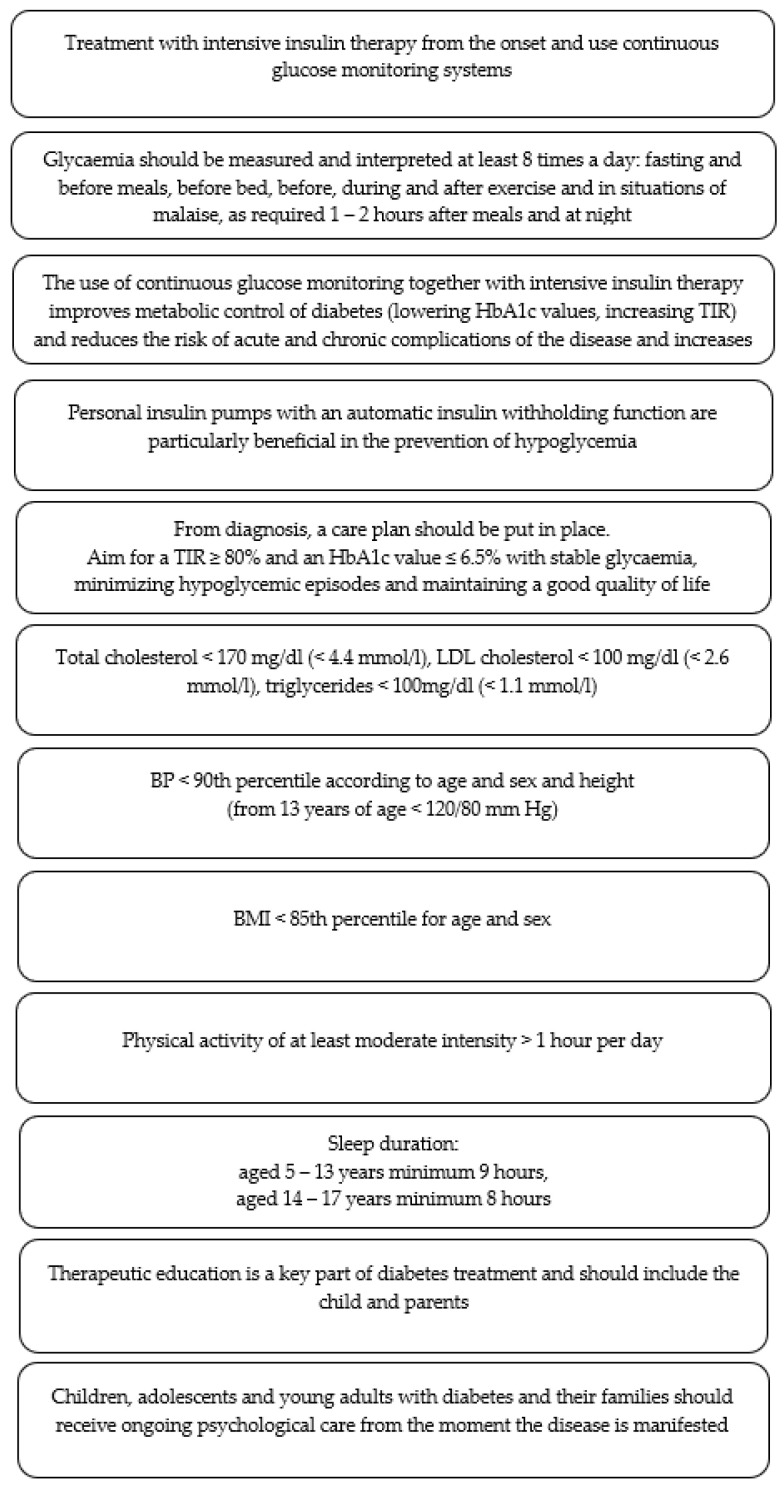
Recommendations and treatment plan goals in children and adolescents with type 1 diabetes according to Polish Diabetology Association 2023 [27].

An equally important medical problem in type 1 diabetes is chronic hyperglycemia. Insulin deficiency, resulting in hyperglycemia, may cause neuropsychological problems manifested in children with type 1 diabetes by a limited speed of information processing, deterioration of conceptual reasoning, and the limited acquisition of new knowledge. If a child becomes ill before the age of 4, the risk of cognitive disorders increases, and with the duration of the disease (6 years and more), attention, general intelligence, speed of perception, long-term memory, and executive functions deteriorate [22]. More and more scientific data draws attention to the harmful effects of hyperglycemia on the structure and function of the brain, especially when the disease begins in the early years of life [25]. Malone et al. believe that chronic hyperglycemia causes greater damage than hypoglycemia to neuronal dendrites, resulting in cognitive impairment [26]. The metabolic control criteria, according to the Polish Diabetes Association 2023, define the requirements to be assessed in the course of treatment. The patient should achieve the following target parameter values for the reduction in the risk of vascular complications: TIR ≥ 80% and HbA1c value ≤ 6.5%, with stable glycemia, minimizing hypoglycemic episodes and maintaining a good quality of life; and LDL < 100 mg/dl, HDL > 40 mg/dl, TG < 100 mg/dl, blood pressure < 90 percentile (from 13 years of age < 120/80 mm Hg), and BMI < 85 percentile. Lifestyle recommendations include physical activity of at least moderate intensity > 1 h per day, and regarding length of sleep, children aged 5–13 years require a minimum of 9 h, and children aged 14–17 years require a minimum of 8 h. A non-smoking lifestyle is also recommended [27]. Compiled recommendations and treatment plan goals in children and adolescents with type 1 diabetes according to Polish Diabetology Association are presented in Figure 2.

In people with type 1 diabetes, with onset at a very young age, chronic complications of diabetic macroangiopathy may develop earlier than in the healthy population and occur in the form of ischemic heart disease, cerebrovascular disease, and lower limb arterial disease. Undoubtedly, both acute complications of diabetes have health consequences, especially when they occur early in life. However, it is not these complications, even if long-lasting, that pose the greatest health risk, but the large amplitude of glycemia achieved, i.e., glycemia instability [19].

### 2.3. Mental Health Problems

Diabetes as a chronic disease changes the life of a child, adolescent, and the entire system in which he or she lives. It interferes with the implementation of the developmental tasks of adolescence, which include achieving new relationships with peers, acceptance of one’s physicality, achieving independence from parents and other adults, achieving economic independence, choosing and preparing for a profession, and preparing for life in marriage and family. Despite the lack of physical symptoms of the disease, people with diabetes have low self-esteem and must regularly check their glucose levels and take insulin, which requires constant possession and use of glucometers, pen-type injectors, insulin pumps, hypoglycemia products, and glycemia monitoring systems. Being a “normal” member of a peer group becomes difficult or even impossible due to the specific nature of nutrition, quantitative and qualitative restrictions, and the timing of meals, which is dependent on insulin kinetics. Life plans, the choice of school and profession, the pursuit of passion, how to spend free time, and the issue of starting a family are subject to verification [22].

An adolescent with diabetes severely experiences parents’ overprotection in striving for self-reliance and independence [28,29]. In chronically ill children, the sense of independence, effectiveness, and coping develops slower due to the enormous involvement of parents, especially when a small child becomes ill. The parent modifies his/her reactions to the child’s requirements, stops setting requirements and necessary boundaries, and believes that self-control activities will be performed better and faster by him/her [29]. Relieving the child of tasks that he/she can perform on their own, not being able to “check” oneself, and presenting demanding attitudes that require society to adapt to the needs of a child with diabetes, deepen the social alienation of teenage patients [28].

According to Małkowska-Szkutnik, teenagers with a chronic disease are more likely to engage in risky behavior, taking into account the fact that engaging in these behaviors translates into significantly better relationships with peers than among chronically ill peers without risky behaviors. In this way, they want to integrate with the group, raise their self-esteem, and build their own identity [28]. Numerous publications present the role of psychological factors in the treatment of diabetes. It has been shown that young people with diabetes have higher levels of anxiety, depression, emotional disorders, psychological difficulties, and eating disorders more often than their healthy peers [29,30,31,32,33].

A poor prognostic factor in the context of these difficulties and inappropriate treatment are adaptation difficulties in the initial period of the disease. In the first four years after diagnosis, depressive symptoms are more severe than in the next five years of illness, and after ten years they become aggravated again [33,34]. Patients with depression and significantly increased anxiety are more often hospitalized due to metabolic decompensation and have insufficient self-control [28]. In a study of American adolescents, the percentage of patients with mild depression was 14%, and severe and moderate depression was found in 8.6%. As in the general population, depression more often affects girls and older teenagers, causes more frequent hospitalizations, and is more likely to lead to mental disorders, especially in patients in whom states of metabolic decompensation with ketoacidosis recur more often. In a study of Polish patients, Zduńczyk observed that there was no difference in the incidence of depression with respect to the level of diabetes self-control. The enormous psychological costs of well-controlled diabetes and the long duration of the disease increase the risk of depression [35,36].

**Figure 3:**
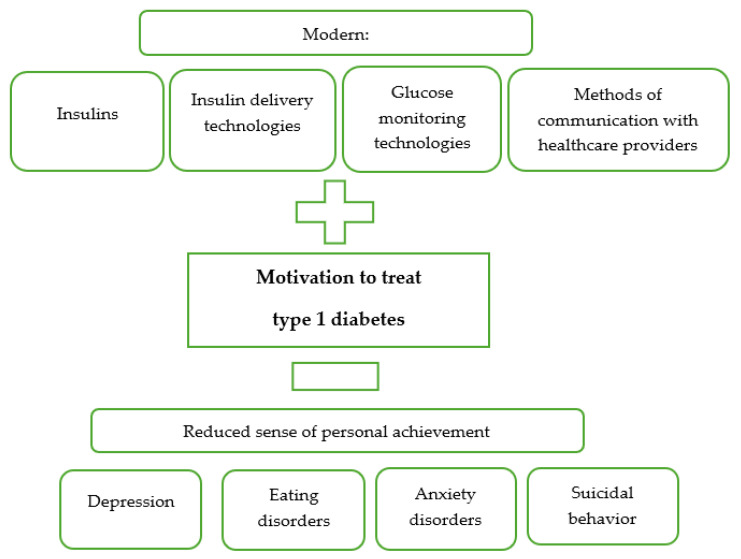
Factors affecting the level of motivation to treat type 1 diabetes.

In addition to depression, mental disorders that develop in the course of diabetes include eating disorders. Constant control of glycemia, the amount of food consumed, and concentration on results including body weight become close to behaviors characteristic of eating disorders, hence diabetics more often than healthy peers experience various eating disorders, including diabulimia, i.e., the intentional reduction or omission of insulin doses [36,37,38]. Girls with diabetes suffer from this condition twice as often as girls without diabetes [39]. Chronic illness, glycemic instability, and constant readiness to make therapeutic decisions mean that patients with type 1 diabetes in the adult population have worse metabolic control of diabetes (HbA1C-8.98%) than patients with type 2 diabetes (HbA1C-7.97%) [40]. Intense self-control activities along with the fear of low blood sugar levels are the cause of the development of a disorder defined by Kamińska et al. as the “therapeutic burnout” syndrome [40,41]. Also, constant care by parents of a chronically ill child creates a risk of overload, known as the caregiver burden with all the health consequences that exceed the caregiver’s adaptative capabilities [42]. In addition to psychological factors that worsen patients’ quality of life and reduce their commitment to self-management of type 1 diabetes, there are also factors that strengthen their motivation. These include modern solutions, which are presented below in Figure 3.

Students with diabetes function worse in the school environment than their healthy peers, and they also have a higher level of school stress. Students with other chronic diseases have a similar level of functioning both in Poland and worldwide [43]. A big problem at school is keeping the disease a secret by children and carers. In the case of diabetes, information about the child’s disease, symptoms warning of hypoglycemia, and how the teacher should intervene during hypoglycemia are necessary factors, and refusing to admit a child to an educational facility due to diabetes is downright reprehensible. The Teacher’s Charter obliges teachers to ensure the safety of students during classes at school [44].

## 3. Effects of Generational Changes

Contemporary young patients with type 1 diabetes are children and adolescents born after 2005. Each subsequent generation creates a certain group identity, i.e., an awareness of who one is as an individual or in a collective [45]. Changes in living conditions are taking place at a great pace, and the next generation of children is surrounded by a completely different culture than the one of their parents. Media technologies determine the lives and identities of young people, and subsequent technological inventions in diabetes management are welcomed with the hope that they will allow them to “forget” about diabetes.

Subsequent generations are increasingly defined not according to the year of birth, but in relation to the media used in a given generation. Getting to know the group facilitates intergenerational communication, allows for mutual understanding, and avoids conflicts [46]. A modern patient of developmental age belongs to the so-called Generation Z and Alpha, which inherited information technologies from the previous Generation Y. A patient from Generation Z lives in times of dynamic development of communication technologies, the growing importance of the Internet, and the acquisition of the new electronic gadgets. This is a generation of “networked” people, referred to as “Digital Natives”, Generation C (Connected Generation), or Net Generation, and they are constantly connected: at home, away from home, while studying, and while resting. A characteristic feature of Generation Z is distraction and the ability to do many things at the same time, especially when pursuing their passions, to which they devote themselves more than to increasing their financial resources [12,47]. This is a generation with blurred boundaries of private and public life, a generation of children and young people constantly present on social media, maintaining the flow of communication on the global network using the mobile phone, which is the focus of all their activities. Young people from this generation are more willing to use images, films, and colors than text; they are more willing to watch a film than to read a book or an article. Twenge notes that the American youth of this generation are immature, growing up slower, and are reluctant to take responsibility for their own decisions and lives, distancing themselves from matters of spirituality and religion, and are reluctant to have children or live in relationships later [48]. Dębski characterized Generation Z as having five features: they are the network generation, they are in love with new technologies, they have low mental well-being, they have high digital competence, and they use mediated communication patterns [49].

Generation Alpha, in turn, consists of children born after 2010, i.e., currently at the age of 14. Representatives of this generation are also referred to as “Generation Glass”, which is characterized by their constant contact with glass interfaces of mobile devices. Kabali et al. indicated that 75% of 375 American children aged six months to four years old have mobile devices, and 96.6% use smartphones or tablets, which they started using before the first year of life [50]. In line with these observations is Bąk’s study on the population of Polish children aged one-and-a-half to six-and-a-half years, indicating that 64% of children use mobile devices, 25% of them daily, with 30% of them being one- and two-year-olds [51]. These devices are used to monitor health status, heart rate, intensity of physical activity, sleep quality, etc. This generation will be the most educated, comfortable, and richest generation, living the longest in the smallest families, according to forecasts. Representatives of this generation tend to consume food products of increasingly poor quality that are highly processed, along with limiting physical activity. Despite unlimited access to the Internet, this generation will be a generation of lonely people experiencing “a new loneliness in closeness” [52]. Considering the characteristics of new generation’s mental issues in the course of diabetes, it seems reasonable to offer psychological assistance to the whole family of the young patient from the diagnosis until the complete adaptation to the condition.

## 4. Modern Technologies in Diabetes

From the point of view of the development of medical technologies supporting the treatment of diabetes, a modern patient receives optimal assistance. Technological progress allows patients to use a continuous glycemia monitoring system with access to real-time continuous glucose monitoring (rtCGM), as well as the ability to track historical glycemia [53]. The system operates by generating a current on the sensor in the presence of the glucose oxidase enzyme, which is inserted into the subcutaneous tissue. The transmitter connected to the sensor collects measurements every 10 s, averages them every 5 min, and transmits the results of the glucose concentration in the interstitial fluid to the recorder, which may be an insulin pump or a separate device [53]. The rtCGM system provides the patient with information about the current glycemia value, the past glycemia profile, and the rate of glycemia changes in the form of trend arrows. The lack of arrows on the recorder provides information to the patient about the stability of glycemia. In turn, their presence indicates the direction and rate of changes in glucose concentration according to the algorithm of the glucose monitoring system used [53]. Diabetes self-control using modern glycemia monitoring technologies gives the patient the opportunity to use information about past, current, and predicted glycemia values in the near future. The use of this data helps patients modify their therapy, including reducing or increasing the insulin dose, skipping a meal, or taking an additional portion of carbohydrates, engaging in physical activity under safe glycemic conditions, and replacing the infusion set, which in turn helps the patient reduce glycemic variability [53]. The safety of therapy, especially in patients in the youngest age groups, is improved thanks to the use of alarm functions in rtCGM devices. The user of the continuous glucose monitoring system can set a threshold and predictive alarm for hypo-and hyperglycemia, an alarm for the rate of increase and decrease in glycemia, and a reminder of method; glucose measurement using flash glucose monitoring (FGM) is available [53]. The FGM sensor measures glucose in the interstitial fluid every minute and saves in the sensor’s memory every 15 min, and by bringing the reader closer to the sensor, the so-called scanner, 8 h of data are transferred to the reader’s memory [54]. Along with the score, trend arrows and a graph of changes over the last 8 h are also displayed. In both monitoring systems, in which the measurement is made in interstitial fluid, there may be a difference in results compared to the blood test, which is due to the physiological delay between these environments. The estimated delay time is about 10–20 min, and after meals with a high glycemic index, up to 40 min [54]. Both CGM and FGM systems can be read using a computer system, and the data contained in them can be analyzed for treatment results. An important criterion for diabetes control is the time spent in the target range (TIR, time-in-range). It is recommended that a type 1 diabetic patient using CGM or FGM should achieve more than 70% of daily readings between 70 and 180 mg/dL, less than 4% of readings below 70 mg/dL, and less than 25% of readings with values above 180 mg/dL [27]. The young patient has the opportunity to send data to the therapeutic team and contact them electronically, thanks to which the patient gains a greater sense of security. The use of monitoring systems and personal insulin pump therapy are recognized tools for improving glycemia (HbA1C, TIR) and reducing the number of episodes of ketoacidosis. The review by Jackowiak-Kamińska indicates that modern technological solutions bring the expected therapeutic effects along with financial and educational support [41].

## 5. Future Challenges

Referring to the outlined profile of a modern teenager, two issues need to be considered—on the one hand, the psychosocial problems arising in the course of diabetes need to be addressed. On the other, education needs to be addressed, not only as a process of education, i.e., transmitting and absorbing content, but also transmitting and acquiring values; this aspect is behind technological development.

A child with diabetes does not feel good about the disease, feels insecure about themselves, and sometimes begins to dislike their own body, and in order to build self-esteem, it is advisable to take additional work on and with oneself as part of psychotherapy [55]. Psycho-education also seems necessary for parents. Feeling guilty for their child’s illness, they forget that they have to care for the child by placing expectations, treating them as healthy, and supporting them when they have temporary crises. Many specialists in Poland and abroad draw attention to the need for and importance of psychological care in diabetes, including the authors of the Polish Clinical Recommendations Polish Diabetes Association 2023, ISPAD (International Society for Pediatric and Adolescent Diabetes), and ADA (American Diabetes Association). Unfortunately, data on the health status and availability of psychological care for patients with diabetes are quite limited, both locally and internationally [39]. Observing the change in the patient’s profile in the context of generational changes, it should be concluded that this care will become a priority challenge, especially in view of its long-term deficits and the deteriorating mental condition of the young generation population. In the ranking of 38 countries assessing the well-being of children and young people (taking into account the suicide rate and level of life satisfaction) prepared by UNICEF, Poland took 30th place [56].

The next challenge will be therapeutic education, which is already a “missing link” [56]. In addition to the staff deficits in the position of a diabetes educator indicated in the report, taking into account the services in the field of therapeutic education in diabetes contracted separately by the National Health Fund, the challenge of the time will be to develop a technological idea that would be a channel of communication with the “networked Glass generation”, and above all, to ensure access for psychological and psychotherapeutic care. Help provided to chronically ill children, such as learning about their feelings, states, and moods, teaching them how to name and express emotions, and relieving tensions without turning them against each other, becomes a way to achieve acceptance of themselves, the disease, and the demands it brings into the life of the entire family. A positive self-image will become the beginning of the path to mature, effectively conducted self-control, which is an expression of care and protection of oneself [29].

## 6. Conclusions

Diabetes is a risk factor for the development of psychological problems in children and adolescents. In these age groups, psychological problems are three times more common compared to healthy peers. The profile of diabetic patients in diabetology is changing, as new generations attempt to achieve maximum success with the least amount of effort. The patients willingly entrust the management of therapy to modern technologies, which resonate with the high digital competence of Generation Z and Alpha. Attention should be paid to the deteriorating mental condition of the young generation, who, in the course of diabetes, are exposed to additional psychological and psychiatric health problems. Prophylactic activities should be performed with patients and their families by monitoring their mental state, providing support, and encouraging the use of professional psychological and psychiatric care. The next generation of patients will need psychological care more than modern technologies, which is why the challenge of the future is to create psychodiabetology centers. Also, healthcare professionals should possess and develop strong motivational dialogue skills. In cooperation with a psychologist, patients should be helped to develop and refine their own coping strategies, build up a sense of self-efficacy for life tasks, and reduce psychosocial stressors skillfully.

## Data Availability

Data used in this study is available on a reasonable request by a corresponding author.
